# How Reliable Is Ki-67 Immunohistochemistry in Grade 2 Breast Carcinomas? A QA Study of the Swiss Working Group of Breast- and Gynecopathologists

**DOI:** 10.1371/journal.pone.0037379

**Published:** 2012-05-25

**Authors:** Zsuzsanna Varga, Joachim Diebold, Corina Dommann-Scherrer, Harald Frick, Daniela Kaup, Aurelia Noske, Ellen Obermann, Christian Ohlschlegel, Barbara Padberg, Christiane Rakozy, Sara Sancho Oliver, Sylviane Schobinger-Clement, Heide Schreiber-Facklam, Gad Singer, Coya Tapia, Urs Wagner, Mauro G. Mastropasqua, Giuseppe Viale, Hans-Anton Lehr

**Affiliations:** 1 Institute of Surgical Pathology, University Hospital Zurich, Zurich, Switzerland; 2 Institute of Pathology, Cantonal Hospital Luzern, Luzern, Switzerland; 3 Institute of Pathology, Cantonal Hospital Winterthur, Winterthur, Switzerland; 4 Institute of Pathology, Cantonal Hospital Graubünden, Chur, Switzerland; 5 Cantonal Institute of Pathology, Liestal Hospital, Liestal, Switzerland; 6 Institute of Pathology, University Hospital, Basel, Switzerland; 7 Institute of Pathology, Cantonal Hospital St. Gallen, St. Gallen, Switzerland; 8 Pathology Institute for Biopsy Diagnostics, Zurich, Switzerland; 9 Medical Laboratory, Pathology, Promed SA, Marly, Switzerland; 10 Institute of Pathology, Municipal Hospital Konstanz, Konstanz, Germany; 11 Institute of Pathology, Cantonal Hospital Baden, Baden, Switzerland; 12 Institute of Pathology, Inselspital, Bern, Switzerland; 13 Pathology, Unilabs Mittelland, Bern, Switzerland; 14 Division of Anatomic Pathology, European Institute of Oncology and University of Milan, Milano, Italy; 15 Institute of Pathology, Centre Hospitalier Universitaire Vaudois, Lausanne, Switzerland; University Medical Centre Utrecht, The Netherlands

## Abstract

Adjuvant chemotherapy decisions in breast cancer are increasingly based on the pathologist's assessment of tumor proliferation. The Swiss Working Group of Gyneco- and Breast Pathologists has surveyed inter- and intraobserver consistency of Ki-67-based proliferative fraction in breast carcinomas.

**Methods:**

Five pathologists evaluated MIB-1-labeling index (LI) in ten breast carcinomas (G1, G2, G3) by counting and eyeballing. In the same way, 15 pathologists all over Switzerland then assessed MIB-1-LI on three G2 carcinomas, in self-selected or pre-defined areas of the tumors, comparing centrally immunostained slides with slides immunostained in the different laboratoires. To study intra-observer variability, the same tumors were re-examined 4 months later.

**Results:**

The Kappa values for the first series of ten carcinomas of various degrees of differentiation showed good to very good agreement for MIB-1-LI (Kappa 0.56–0.72). However, we found very high inter-observer variabilities (Kappa 0.04–0.14) in the read-outs of the G2 carcinomas. It was not possible to explain the inconsistencies exclusively by any of the following factors: (i) pathologists' divergent definitions of what counts as a positive nucleus (ii) the mode of assessment (counting vs. eyeballing), (iii) immunostaining technique, and (iv) the selection of the tumor area in which to count. Despite intensive confrontation of all participating pathologists with the problem, inter-observer agreement did not improve when the same slides were re-examined 4 months later (Kappa 0.01–0.04) and intra-observer agreement was likewise poor (Kappa 0.00–0.35).

**Conclusion:**

Assessment of mid-range Ki-67-LI suffers from high inter- and intra-observer variability. Oncologists should be aware of this caveat when using Ki-67-LI as a basis for treatment decisions in moderately differentiated breast carcinomas.

## Introduction

The last twenty years have witnessed a marked decline in breast cancer mortality, largely due to earlier diagnosis, a better understanding of the disease, and the advent of ever more effective adjuvant treatment options [1], [2]. This progress in adjuvant systemic therapy has led to consensus recommendations proposing adjuvant therapy to virtually all breast cancer patients [3]. At the same time, we have to acknowledge that in order for some patients to benefit from these adjuvant therapies, many others are treated with little or no benefit – except for untoward effects [4]. Chemotherapy targets proliferating tumor cells, high proliferative activity of breast carcinomas predicts response to chemotherapy [5], [6] as well as endocrine therapy [7], and a drop in proliferative activity after short-term neoadjuvant endocrine therapy predicts prolonged disease-free survival [8].

Proliferative activity has historically been assessed by counting mitotic figures at high magnification as well as by immunohistochemical detection of Ki-67, a nuclear protein that is expressed in proliferating cells [9]. In the light of the important prognostic and predictive role of proliferative activity in breast cancer, it is not surprising that immunohistochemical detection of Ki-67 using the MIB-1 antibody has gained increasing importance in routine breast cancer diagnosis and has recently been recommended by the St.Gallen consensus conference [10], [11]. MIB-1-based proliferative fraction of breast carcinomas thus can be taken into consideration when defining an adjuvant treatment plan for cancer patients. This decision is eather straightforward in large, poorly differentiated carcinomas showing many (atypical) mitotic figures or in small, highly differentiated carcinomas where only scarce mitotic figures are found. These cases typically show a high MIB-1 labelling index (LI) of more than 30% and a low MIB-1-LI of less than 5%, respectively. In contrast, the putative chemotherapy benefit may be more difficult to judge in moderately differentiated carcinomas. Recent gene signature data suggest that these G2 carcinomas can be separated into two categories, with biological behaviors similar to either G1 or G3 carcinomas [12]. This separation of G2 carcinomas into “pets” and “raptors” is defined by genes that drive tumor proliferation and could be reproduced with high statistical power using immunhistochemical detection of Ki-67 [11]–[15]. This has recently been designated the “tip effect “ that MIB-1-LI may play in moderately differentiated carcinomas with indefinite prognosis [15].

For these reasons, oncologist have high expectations in the MIB-1-LI, notably for patients with G2 carcinomas. Yet, how reliable is the immunohistochemical technique and how reliable are pathologists in assessing MIB-1 based proliferative activity in individual patients? The present study was designed as a quality control measure within the Working Group of Breast- and Gynaecopathologists in the Swiss Society of Pathology to investigate how accurate and reliable Ki-67 fractions are in moderately differentiated carcinomas.

## Materials and Methods

Cases were selected from the archives of the Department of Surgical Pathology, University Hospital Zürich. The study was submitted to the local Instituitional Review board and complied with institutional guidelines. Immunohistochemical stains were created from paraffin blocks in a strictly anonymized fashion. No patient consent was required.

The study evolved in three steps. Between each step, all pathologists participated in joint discussions of the results and the study protocol.

### Step one

Ten random cases of invasive breast carcinomas (two G1 carcinomas, five G2 carcinomas, and three G3 carcinomas) [16] were immunostained with MIB-1 according to standard protocols (DAKO M7240, 1∶20) using the Ventana automated Benchmark staining system (Ventana, Tucson, AZ) and sent to five pathologists (central immunostain). In addition, one unstained slide of each case was provided that the participating pathologists were asked to immunostain in their own laboratories (local immunostain). They were asked to quantify MIB-1-LI by rough estimation (eyeballing) and by counting MIB-1 positive tumor cells among a total of 2000 cells. No guidelines were given as to where (within the tumor) to count and what exactly constitutes a MIB-1 positive nucleus. Also, it was left to the pathologists to decide whether they first counted MIB-1 positive cells or whether they first estimated their percentage, as long as they made sure that the result of one quantification technique did not affect the results on the corresponding other technique. This was done by first eyeballing all the cases in random order and then counting all the cases in random order, or vice versa.

### Step two

**Figure 1:**
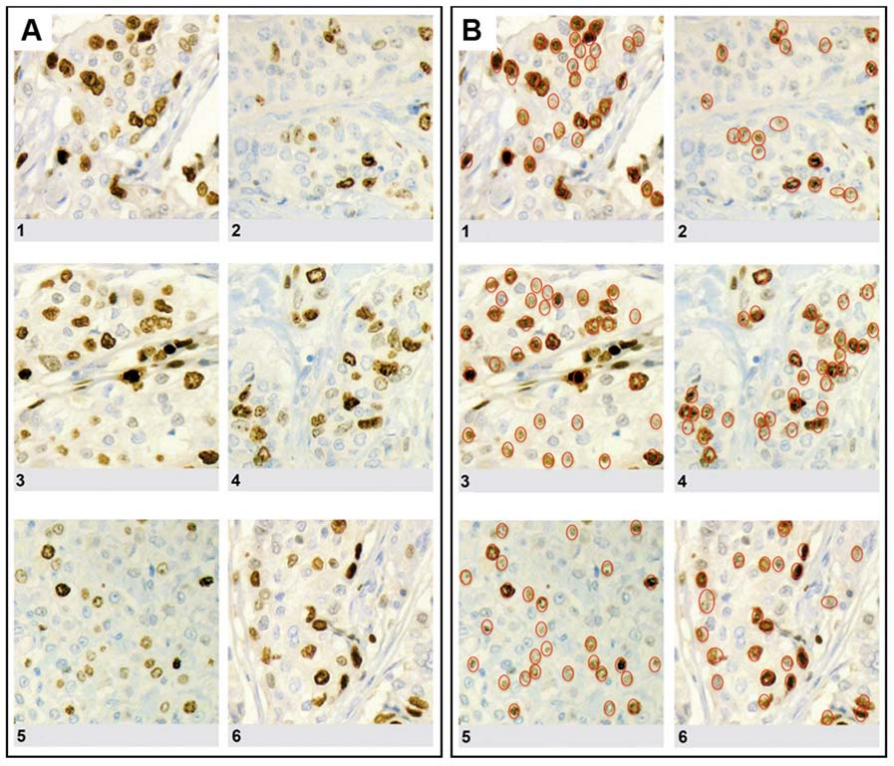
Color plate with 6 color images of MIB-1 immunostains of breast carcinomas. A: Six 150 µm×150 µm fields from MIB-1 immunostains of moderately differentiated breast carcinomas were selected so as to cover a wide spectrum of immunostaining intensities, each image containing some intensely stained nuclei, some clearly negative nuclei, but also a spectrum of intermediate immunostaining intensities. The participating pathologists were asked to count and circle the number of MIB-1 positive and MIB-1 negative nuclei for each image. B: The right part of the figure shows the same plate once more, only that this time those nuclei were circled that were considered MIB-1 positive by all investigators upon joint discussion. This plate was provided to the participants of the study along with the slides for step 3, with the aim to further reduce interobserver variabilities in MIB-1-LI results.

Three G2 breast carcinomas, showing a rather uniform proliferative activity, were sent to 15 pathologists all of whom regularly attended the meetings of the Working Group. For each case, participants received one slide stained in Zürich (central immunonstain) and one empty slide which he/she immunostained in their own laboratory. In addition, an envelope was provided, which had to be opened only after each pathologist had evaluated the MIB-1-LI on the six slides in areas of the tumor that he/she had selected him/herself. This envelope contained a digital picture of each slide with five circled areas, in which MIB-1-LI had to be assessed one more time. Participants were asked to provide MIB-1-LI results by eye-balling and by counting positive tumor cells among 500 tumor cells. In addition, each participant received a letter-sized “intensity” plate containing 6 color images of MIB1-stained slides (final magnification: 150 µm×150 µm, **Figure 1A**), on which he/she was asked to count all MIB-1-positive and all -negative tumor cells.

### Step three

With the aim to improve reproducibility of MIB-1 read-outs, we repeated step two four months later, but this time provided clear guidelines (i) where within a tumor MIB-1-LI should be analysed (in the tumor periphery, avoiding hot-spots), and (ii) what exactly constitutes a MIB-1 positive nucleus. To this end, the “intensity” plate was distributed, on which all positive nuclei were circled upon consultation with Pr. Giuseppe Viale (**Figure 1B**). To the three G2 carcinomas used in step two, we added three new carcinomas (G1, G2, G3).

### Statistical analysis

Statistical analyses were performed using online calculators, http://amchang.net/StatTools/CohenKappa_Pgm.php for Fleiss's and Cohen's Kappa, and http://www.wessa.net/rwasp_spearman.wasp for Spearman linear correlations. To calculate Kappa values, MIB-1-LI data were stratified into a three-tier scoring system, using arbitrary cut-offs of 8% (G1 vs. G2) and 15% (G2 vs. G3). Wilcoxon tests were performed to test for deviation of individual participants from the group mean.

## Results

### Step one

**Figure 2:**
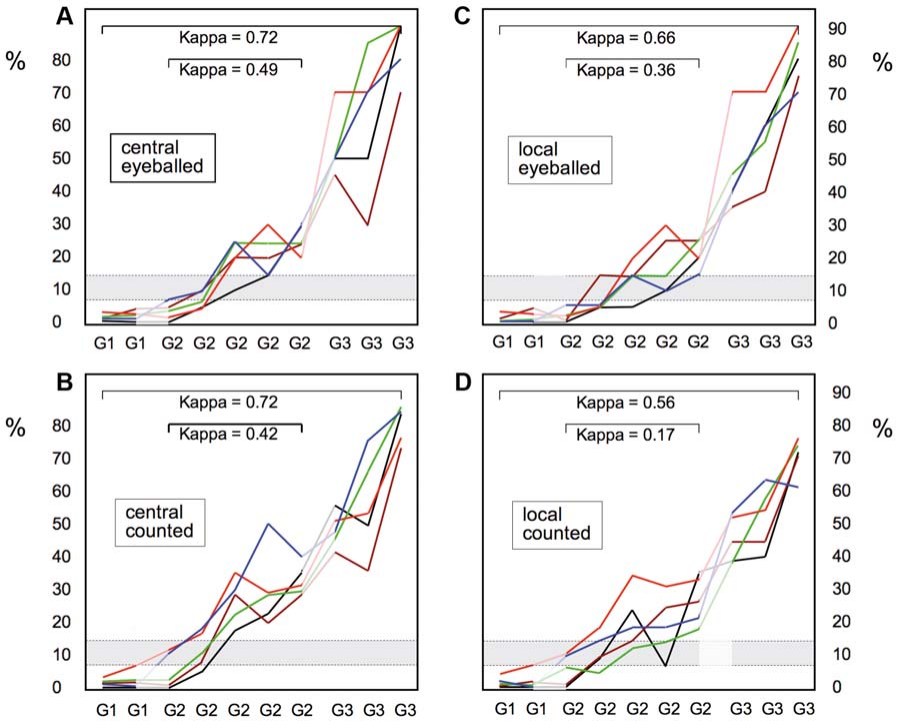
MIB-1-LI results of ten breast carcinomas read by five pathologists (represented by lines in different colors): The left panels (A,B) depict the results obtained in centrally immunostained slides and the right panels (C,D) the results on locally immunostained slides. The upper panels (A,C) are eyeballed and the lower panels (B,D) counted data. Despite marked variability between the five observers, MIB-1-LI results were all below 8% for the two G1 carcinomas and above 30% for the three G3 carcinomas. Note that MIB-1-LI results varied considerably for the five moderately differentiated carcinomas. Shown in grey is the zone deliminated by the 8% and 15% cut-offs used for calculating the kappa scores for interobserver correlations.

**Figure 3:**
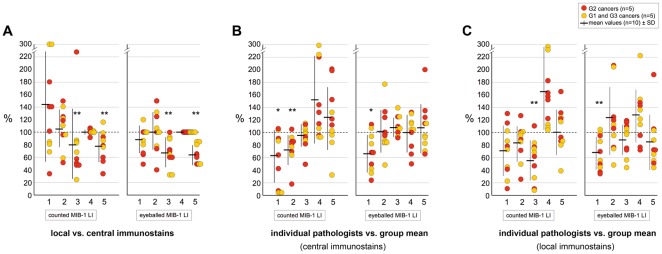
Variability of MIB-1-LI results (ten carcinomas, five pathologists): MIB-1-LI data obtained by five pathologists on immunostained slides of five moderately differentiated breast carcinomas (G2, red circles) and five G1 and G3 carcinomas (yellow circles), once assessed by counting the number of MIB-1 positive nuclei among 2000 tumor nuclei (left graph) and once by eyeballing the LI (right graph). Mean values over the ten carcinomas are shown in horizontal bars and standard deviations in vertical bars. A. local vs central immunostains: shown are fractions of MIB-1-LI results on locally immunostained slides over the results obtained on centrally immunostained slides. The observers 3 and 5 significantly underrated MIB-1-LI on the slides immunostained in their own laboratory when compared to the central laboratory (**P<0⋅01 Wilcoxon). B,C. individual pathologist vs group mean: shown are fractions of MIB-1-LI results by individual pathologists over the mean value calculated for the group of fibve pathologists for each individual carcinoma. Note that the observer 1 tended to significantly underrate MIB-1-LI when compared to the mean values obtained by the entire group of five pathologists (*P<0⋅05 and **P<0⋅01 Wilcoxon). Also note that the deviations from the group mean values tended to be smaller for the eyeballed than for the counted data.

MIB-1-LI results were all below 8% for G1 and above 30% for G3 carcinomas. However, interobserver variability was substantial for the G2 cases, values ranging between 5 and 30% for the same cases (**Figure 2**). We found good to very good kappa values for interobserver correlations over the entire group of ten carcinomas (G1–G3, 0⋅56–0⋅72), but only poor to moderate correlations when the analysis was limited to the five G2 carcinomas (kappas 0⋅17–0⋅49, **Figure 2**). We calculated for each pathologist and each tumor the fraction of MIB-1-LI results over the mean MIB-1-LI of the whole group of five pathologists (**Figure 3B,C**). Significant deviations from group mean values were found for two of the pathologists (**Figure 3B,C**). The severity of the deviation of MIB-1-LI results from group mean values tended to be more pronounced for the data obtained by counting than by eyeballing (**Figure 3B,C**).

To assess the impact of the immunostaining technique on MIB-1-LI values, we expressed the values obtained by each pathologist in local immunostains as fraction over central immunostains. We identified two laboratories in which the local immunostain yielded significantly lower values than the central laboratory (**Figure 3A**). Kappa values showed good to very good intralaboratory correlations. As expected, kappa values were higher for the ten carcinomas then for the five G2 carcinomas (**Table 1**).

**Table 1:** Kappa values for rating scores.

	G2-carcinomas (n = 5)					G1–G3 carcinomas (n = 10)				
pathologist	1	2	3	4	5	1	2	3	4	5
central vs. local immunostains										
eyeballed data	0.76	0.76	0.40	1.00	0.28	0.89	0.89	0.69	1.00	0.68
counted data	0.40	0.76	0.76	1.00	0.54	0.69	0.89	0.89	1.00	0.87
eyeballed vs. counted data										
central immunostains	0.62	1.00	0.78	0.54	0.21	0.80	1.00	0.89	0.78	0.66
local immunostains	0.17	1.00	1.00	0.29	0.00	0.60	1.00	1.00	0.67	0.49

### Step two

**Figure 4:**
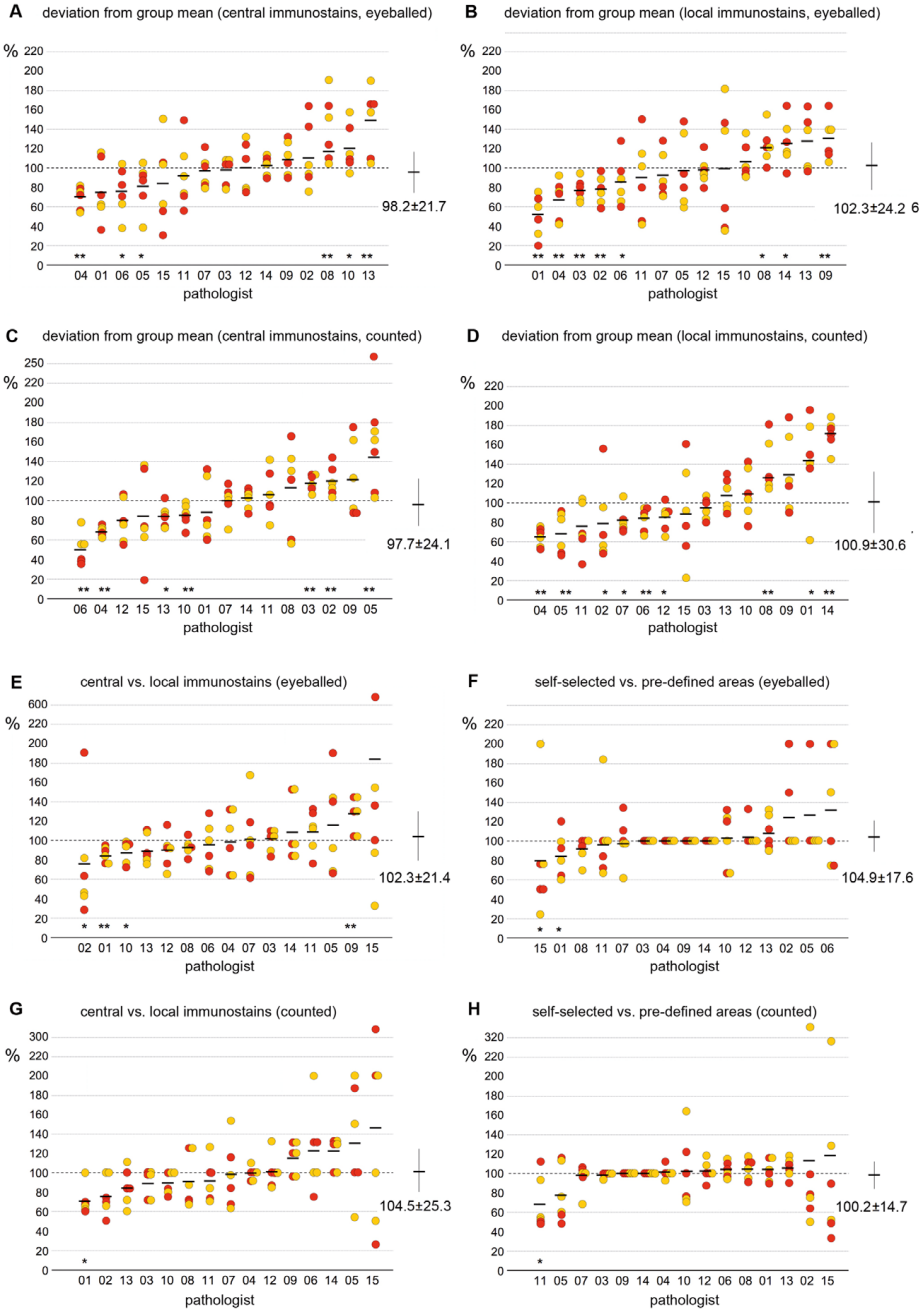
Variability of MIB-1-LI results (three carcinomas, 15 pathologists): MIB-1-LI data obtained by 15 pathologists on immunostained slides of three moderately differentiated breast carcinomas, assessed either by eyeballing the LI or by counting the number of MIB-1 positive nuclei among 500 tumor nuclei. A–D Interobserver variabilities of MIB-1-LI results: Shown are the fraction of MIB-1-LI results by individual pathologists over the mean value calculated for the entire group of 15 pathologists for this same carcinoma. Red and yellow circles indicate results obtained in self- selected and predefined areas, respectively. Mean values are shown in horizontal bars. Shown are the results obtained on centrally immunostained slides (A,C) and on locally immunostained slides (B,D). Several pathologists systematically under-rated (pathologists 4,5 and 6) or over-rated MIB-1-LI (pathologists 8,14) when compared to the mean values obtained by the entire group of 15 pathologists (*P<0⋅05 and **P<0⋅01 Wilcoxon). Note that for the entire group of 15 pathologists, the standard deviations around the mean values were smaller when MIB-1-LI were eyeballed (A,B, SD = 21⋅7 and 24⋅2) as compared to the data that were obtained by counting MIB-1 postive nuclei (C,D, SD = 34⋅5 and 30⋅6). E,G impact of immunostaining technique: shown are for each carcinoma the fraction of MIB-1-LI data on locally immunostained slides over those read on centrally immunostained slides. Red and yellow circles indicate results obtained in self- selected and predefined areas, respectively. Mean values are shown in horizontal bars. The observers 1 and 2 significantly under-rated MIB-1-LI on the slides immunostained in their own laboratory when compared to the central laboratory, suggesting that their immunostaining techniques yielded fainter results than the one of the central laboratory (*P<0⋅05 and **P<0⋅01 Wilcoxon). Note that for the entire group of 15 pathologists, the standard deviations around the mean values were slightly smaller when MIB-1-LI were eyeballed (E, SD = 21⋅4) as compared to the data that were obtained by counting MIB-1 positive nuclei among 500 tumor cells (G, SD = 25⋅3). F,H impact of the tumor area: shown are for each carcinoma the fraction of MIB-1-LI read-outs in areas that each pathologist had selected his/herself over read-outs repeated in areas that had been pre-selected by the principal investigator. Red and yellow circles indicate results obtained in centrally and locally immunostained slides, respectively. Mean values are shown in horizontal bars. For four of the 15 pathologists, there was a near perfect match between self-selected and pre-defined areas within the tumor. Overall, there was a very small variability for the entire group of 15 pathologists.

The study was then extended to 15 pathologists who were asked to assess MIB-1-LI on three G2 carcinomas. Expressing each pathologist's MIB-1-LI values as fraction over the group mean value, we found significant deviations from mean values for several pathologists (**Figure 4A–D**). As in step 1, the severity of the deviation of MIB-1-LI values from mean values was more pronounced for the results obtained by counting than by eyeballing. This is also reflected by the larger standard deviations from the mean values for counted than for eyeballed data. Using kappa statistics, we found very poor interobserver correlations between the 15 pathologists (15 pathologists = raters, three tumors = subjects, three grades = categories). The data were slightly more consistent on centrally stained slides (kappas 0⋅04–0⋅14) than on locally stained slides (kappa 0⋅01–0⋅04), but no difference was seen by pre-defining the field of interest or by counting MIB1-positive nuclei.

The immunostaining techniques applied by the central and the peripheral laboratories are shown in **Table 2**. All but one institution used one of four DAKO clones, in dilutions from ready-to-use to 1∶600. Pretreatments include pressure heat, pressure cooking as well as EDTA and CC1 antibody retrieval solutions. To assess the impact of the immunostaining technique on the MIB-1-LI read-outs, we expressed MIB-1-LI values as fraction of local immunostains over central immunostains (**Figure 4E,G**). We identified two laboratories, in which the local immunostaining technique yielded significantly lower MIB-1-LI values than central immunostains and one laboratory that yielded higher values. These differences were seen both in the analyses of the eyeballed and the counted data. Kappa statistics showed very good correlations between central and local immunostains for all three tumors, when based on MIB1-data assessed by eyeballing (0⋅94, 0⋅82, and 0⋅71 for cases 1, 2, and 3, respectively) and, to a somewhat lesser extent, by counting (0⋅93, 0⋅61, and 0⋅43).

**Table 2:** Laboratory protocols for Ki-67 immunostains in the 12 participating pathology institutes.

Institution	Antibody/Clone	Pretreatment	Dilution	Detection System
1	DAKO/MIB-1	30 min, EDTA, pH 9	1∶100	Leica Bond Max
2	DAKO/M7240	30 min, EDTA, CC1	1∶50	Benchmark
3	DAKO/KI-67	90 sec,cooking, pH 9	1∶500	Autostainer Plus
4	DAKO/M7240	30 min, standard CC1	1∶40	Benchmark
5	Cell Marque/KI-67 (SP6)	90 sec, pressure heat	1∶100	Benchmark
6	DAKO/KI-67	30 min, pressure heat	1∶500	Leica Bond Max
7	DAKO/KI-67	90 sec, cooking	1∶600	Autostainier Plus
8	DAKO/M7240	30 min, CC1 standard	1∶20	Benchmark
9	DAKO/KI-67	30 min, ER1 buffer, pH 6	1∶50	Bond Diluent
10	DAKO/MM1	30 min, ER2 buffer	Ready to use	Leica Bond Max
11	DAKO/KI-67	30 min, EDTA, pH 9	1∶200	Leica Bond Max
12	DAKO/M7240	30 min, CC1 standard	1∶40	Benchmark

When expressing the MIB-1-LI values obtained in self-selected over values in pre-defined areas, we identified three pathologists who apparently selected fields of interest that were significantly less proliferative than the pre-defined areas (**Figure 4F,H**). However, this phenomenon depended on the mode of MIB-1 assessment. For two pathologists (N°1 and N°15), underestimation of MIB-1-LI was seen only for eyeballed data, and for one pathologist (N°11), the underestimation was only seen for counted data. Kappa statistics showed good to very good correlations between self-selected and pre-defined areas for all three tumors, both for eyeballed (0⋅83, 0⋅83, and 0⋅69 for cases 1, 2, and 3, respectively) and for counted values (0⋅61, 0⋅83, and 0⋅83).

MIB-1-LI data as assessed by eyeballing showed a good correlation with data assessed by counting MIB-1 positive nuclei: kappa values for the three tumors were 0⋅77, 0⋅61, and 0⋅60 for cases 1, 2, and 3, respectively, for centrally imunostained slides, and 0⋅65, 0⋅79, and 0⋅64 for locally immunostained slides.

**Figure 5:**
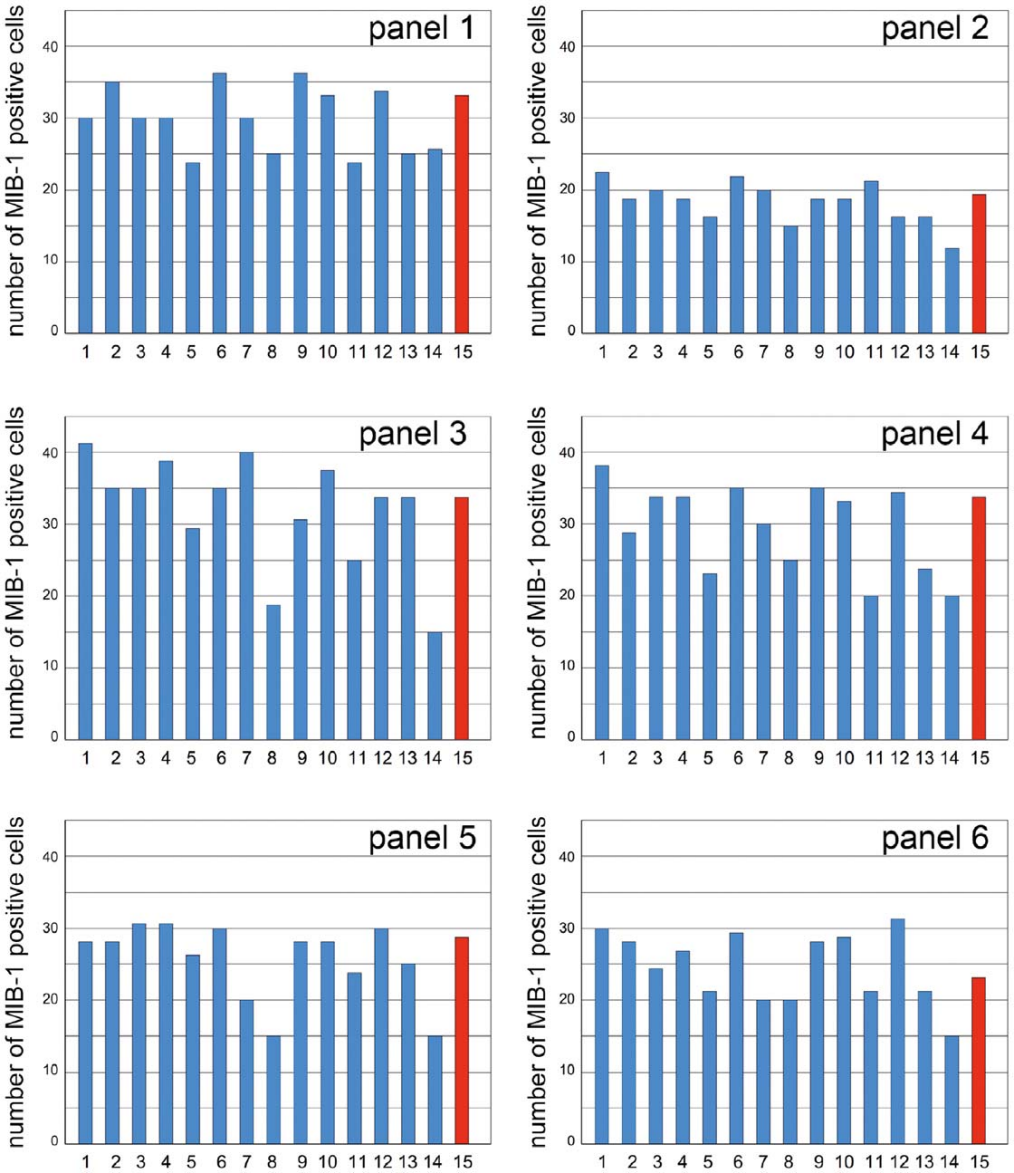
How does a MIB-1 positive nucleus look like? MIB-1-positive cells counted by the 15 pathologists on the 6 high power fields shown in figure 1A. The 16th bar in red shows the counts provided by Pr. Giuseppe Viale who helped us standardize which nuclei should be considered MIB-1-positive in the right panel of **figure 1 (1B)**. Note that several pathologists counted only those nuclei that were intensely immunostained (pathologists 8,11,14), while most included in their count also most faintly immunostained nuclei. Note that the results were very homogeneous for the 6 different high power fields.

**Figure 6:**
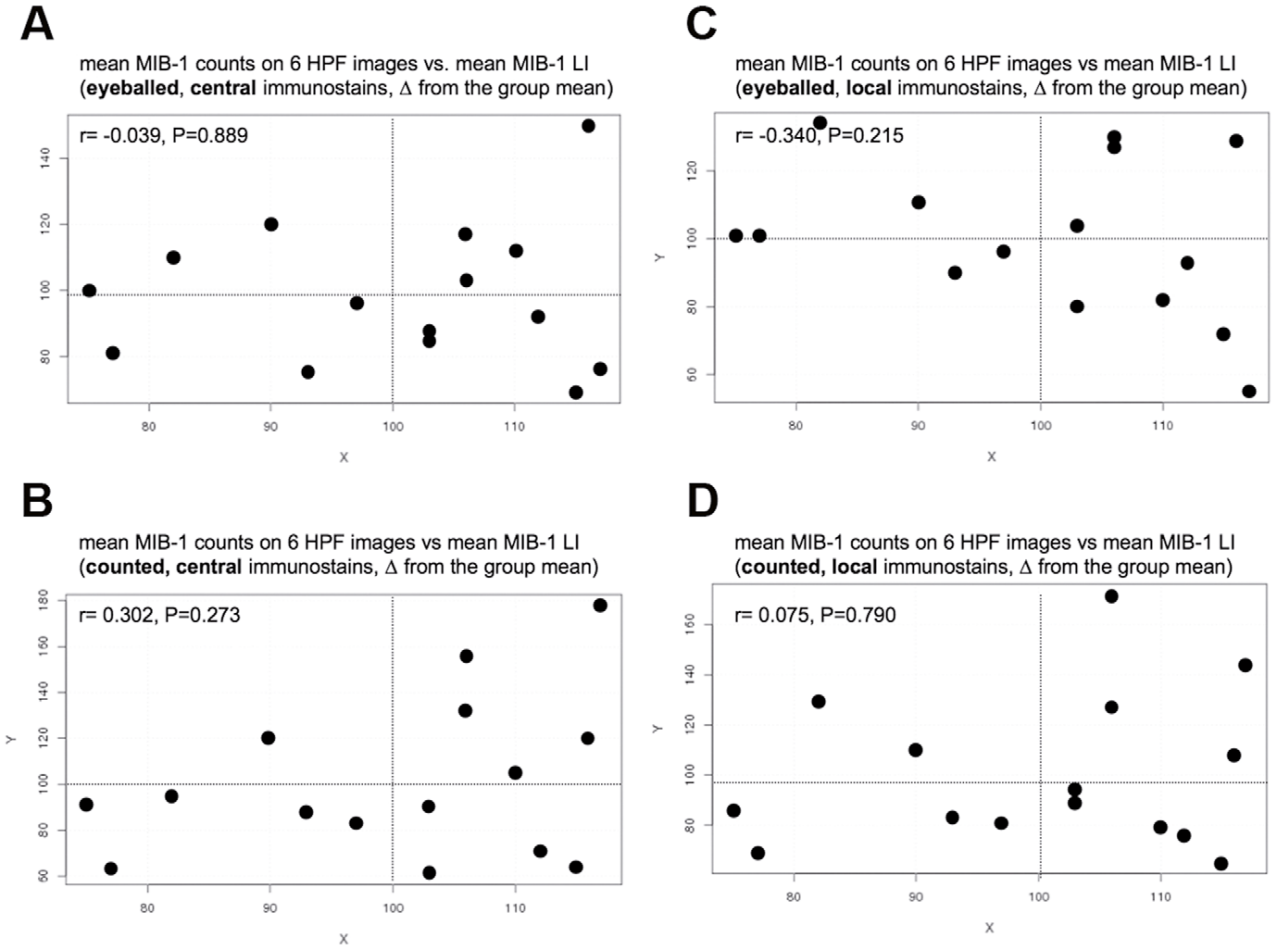
Absence of linear correlations between the MIB-1 counts obtained in the 6 high power fields (figure 1) and the MIB-1-LI results obtained on the three moderately differentiated carcinomas (figure 4). The linear correlations were calculated separately for data obtained by eyeballing (upper panels, A,C) and by counting (lower panels, B,D) and for data obtained on centrally immunostained slides (left panels, A,B) and on locally immunostained slides (right panels, C,D). This analysis was done in order to test the hypothesis that under-raters (i.e. pathologists 4,5, and 6 in **figure 4**) considered only very intensely immunostained nuclei as positive and vice versa. However, this hypothesis was proven wrong: we found no significant linear correlations between the MIB-1-LI results on the three carcinomas and the number of MIB-1 positive nuclei counted by the same 15 pathologists in the 6 panel plate.

We next asked the question whether the differences in MIB-1-LI results between pathologists might be due to different individual perceptions of what a MIB-1-positive nucleus looks like. We assumed that those pathologists who consistently read higher MIB-1-LI values should have a lower threshold, considering even faintly stained nuclei as positive, and vice versa. All 15 pathologists were hence asked to count positive nuclei in six images of MIB-1 immunostains (**Figure 1A**), and the distribution of MIB-1 counts was comparable to the distribution of values obtained on the slides, some pathologists counting considerably fewer nuclei than others (**Figure 5**). However, when we performed linear regression analyses of mean MIB-1-LI values rendered on the microscope slides by each individual pathologist against his/her mean counts rendered on the 6 color plates, we found no significant correlation: Rho values were below 0⋅30 (Spearman; **Figure 6**), suggesting that the interobserver variability could not be explained by differences in threshold levels for what each pathologist consider as a MIB-1 positive nuclei.

### Step three

**Figure 7:**
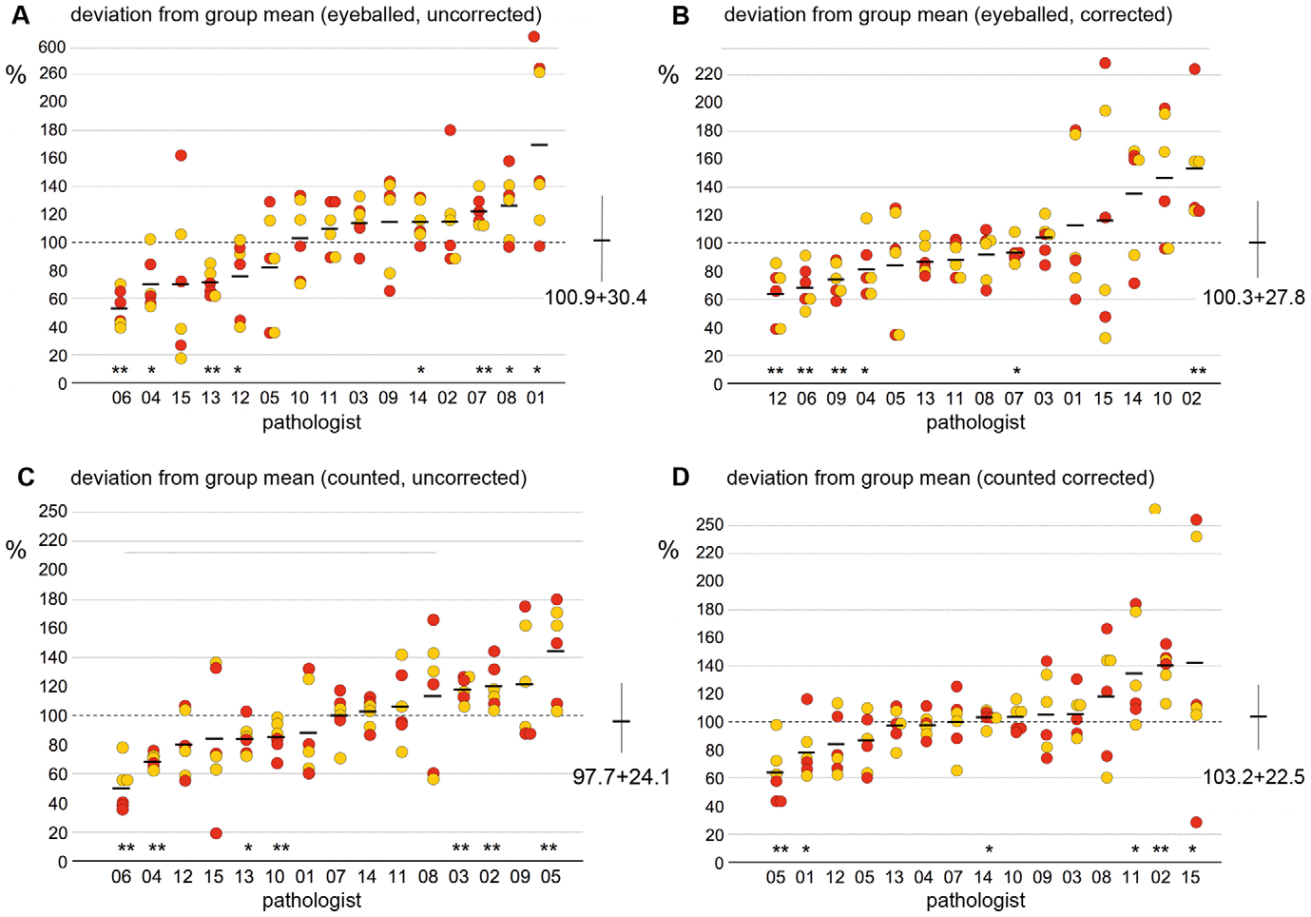
Interobserver variabilities in MIB-1-LI results, four months later: MIB-1-LI data obtained by 15 pathologists on centrally immunostained slides of three moderately differentiated breast carcinomas, assessed by eyeballing (A,B) and counting (C,D). Shown are for each pathologist the fraction of MIB-1-LI results over the mean value calculated for the entire group of 15 pathologists. Red and yellow circles indicate results obtained in self- selected and predefined areas, respectively. Mean values are shown in horizontal bars. The interobserver variabilities were comparable to the results obtained four months prior on the same slides (see **figure 4B**). The data shown in the right panels (B,D) were then modified by a “correction factor” for each individual pathologist. This correction factor was calculated from the results obtained on three independent carcinomas, see Table 3). Yet, this correction factor could not reduce the high interobserver variability, as evidenced by only slight reductions of the standard deviations (27⋅8 vs 30⋅4 for eyeballed and 22⋅5 vs 24⋅1 for counted data).

Four months after step 2, the same 15 pathologists were asked to assess MIB-1-LI once more on the same three G2 carcinomas that had been used for step 2, as well as on three new breast carcinomas (G1, G2, G3). Interobserver variability, as calculated in analogy to step 2, was not improved: kappa values were even smaller than those in step 2 (kappa 0⋅01–0⋅04) and the standard deviations of the mean values were in the same range as the ones in step two (**Figure 7A,C**).

In the first and the second step of this study, we found that certain pathologists tended to systematically over- or underrate MIB-1 LI. We hence tested whether a «correction factor» could be established by calculating the deviation of each individual pathologist's MIB-1-LI results from the group mean for the three new carcinomas (**table 3**). However, when applying this correction factor to the results of the initial three carcinomas, the interobserver correlation did not improve, as evidence by largely identical standard deviations from the mean values (**Figure 7B,D**).

**Table 3:** Step three, Ki-67 mean values (6 carcinomas, 15 pathologists).

	central immunostains			
	assessment by eyeballing		assessment by counting	
	self-selected areas	predefined areas	self-selected areas	predefined areas
Case 1 (%)	13.9±6.8	14.2±4.5	13.7±4.4	14.6±3.6
corrected	15.0±6.9	14.9±5.8	14.3±4.6	13.7±4.3
Case 2 (%)	11.3±6.5	11.5±6.6	10.7±4.3	11.2±3.6
corrected	11.1±5.2	11.3±5.3	11.2±2.9	11.6±2.8
Case 3 (%)	15.5±4.3	12.9±3.9	15.0±6.5	14.2±5.2
corrected	16.9±8.5	13.2±4.3	12.5±5.0	13.7±4.2
Case 4 (%)	3.4±2.0	3.1±2.0	3.5±2.1	2.7±1.3
Case 5 (%)	12.5±4.3	12.0±4.6	12.6±4.6	12.2±3.9
Case 6 (%)	22.9±6.4	23.1±4.8	25.6±5.2	25.1±6.2

**Figure 8:**
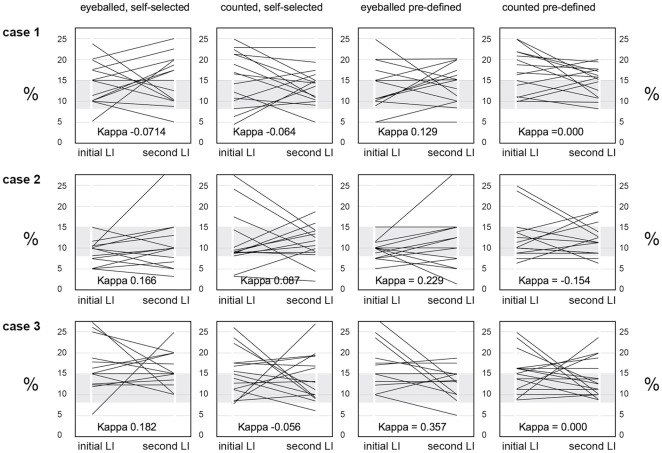
Intra-observer variabilities of MIB-1-LI results. Shown are in each panel the MIB-1-LI results for each pathologist during step 2 of the study (left end of each line, one line representing the values obtained by one pathologist) and during step 3, 4 months later (right end of each line). The grey zone delineated the 8% and 15% cut-offs used for calculaing Kappa values. The three rows represent the results obtained for the three different carcinomas (cases 1–3). The left two columns of panels show the results obtained by reading MIB-1-LI in areas that the pathologists selected themselves (self-selected) and the panels in the two right columns the values assessed in pre-defined areas (pre-defined). In each pair of columns, values were assessed by eyeballing (left) and by counting (right). As expected, Kappa values for intraobserver variability were slightly better in pre-defined areas than in self-selected areas, and, in analogy to the interobserver variability studies, better in eyeballed than in counted MIB-1-LI read-outs.

To assess the intra-observer variability of the MIB-1-LI values, we plotted for each pathologist his/her values obtained during step 2 and again four month later (**Figure 8**). For some tumors and some pathologists, MIB-1-LI values migrated from values between 5–10% to values above 25% and vice versa. We also calculated kappa scores using the same cut-off values of 8% and 15% that were used in the prior analyses. As shown in **figure 8**, the kappa scores indicated very poor consistency. They were slightly higher when MIB-1-LI were assessed in pre-defined areas within the carcinomas (vs. self-selected areas), and when MIB-1-LI was assessed by eyeballing (vs. counting). However, the chance that a breast carcinoma which had been assigned an intermediary score in step 2 of the study received the same intermediary score once again by the same pathologist a few months later was between 5/15 and 9/15 under the various conditions, barely superior to throwing dice (expected value: 5/15).

## Discussion

The principal outcome of this quality control effort is that interobserver variability of MIB-1 labeling index in breast carcinomas is (i) more problematic than we had expected, (ii) not easily explained by obvious confounders such as the immunostaining technique and the selection of the tumor area, (iii) not reduced by (meticulous) counting versus (rapid) eyeballing, and (iv) not improved by efforts to standardize what exactly are MIB-1 positive nuclei and where and how to count them.

*… interobserver variability of MIB-1 labeling index in breast cancer is more problematic than we had expected.* At first sight, assessing MIB-1-LI appears as a simple task. We do it every day, and even when we integrate various opinions on individual tumors around the multiheaded microscope, we seem to quickly arrive at a number that everyone can agree with. Kappa values for interobserver consistencies for MIB-1-LI in different tumors are usually in the range of 0⋅60–0⋅85, suggesting a good reliability of this marker [13], [17]–[20]. Indeed, in the first step of our study, we obtained similarly “good” kappa values (0⋅56–0⋅72) on the ten breast carcinomas whose degree of differentiation ranged from G1 to G3 carcinomas (**figure 3**). However, when only the five G2 carcinomas were considered, Kappa values fell to values between 0⋅17 and 0⋅49, reflecting poor to moderate agreement at best. This is particularly worrisome because these are the carcinomas for which oncologists hope to obtain guidance with our MIB-1-LI values for their chemotherapy decisions [13], [14]. It is hence in this group of moderately differentiated carcinomas where our capacity to reliably diagnose MIB-1-LI for individual patients is put to test.

*…. not easily explained by obvious confounding factors.* Even though we found that immunostaining techniques, including pre-treatment protocols and antibody dilutions varied tremendously between laboratories, the comparison between MIB-1-LI on centrally and locally stained slides showed only small and inconsistent variations, both in the initial study with five observers and in the main study with 15 observers. This finding does not support the proposition that individual MIB-1 cut-offs should be established for each individual laboratory in order to reflect differences in immunostaining techniques [21]. We then asked whether the choice of the **field of interest** is important. This is in fact the standard “excuse” when MIB-1-LI read-outs cannot be reproduced, for instance when we review our colleagues' cases for the weekly tumor board. We adopted the view that proliferative activity should be assessed in the proliferating active tumor periphery and that - in contrast to earlier proposals [22] - hotspots should be avoided. If the choice of the field of interest were to have exerted a marked impact on interobserver variability, we should have found significant deviations of MIB-1-LI between self-selected and pre-defined fields of interest for several of the observers. However, this was not the case. We found only small variations between self-selected and pre-defined fields of interest. This observation is supported by reports that variations between random fields selected from within the growth zone of invasive breast carcinomas are usually quite small [22]–[24].

*…not reduced by (meticulous) counting versus (rapid) eyeballing.* We have often disputed whether MIB-1-LI should be **counted** (and if yes, should we count 500, 1000, or even 2000 tumor cells) or whether it is just as good to simply **eyeball** the labeling index. When analysed side by side, both techniques yield similar results [25]. Yet, none seems to have ever systematically analyzed which of the two methods yields more reproducible results. This may be because there is no “truth” to compare with, or because the answer seemed so very obvious: of course, counting ‘must be’ better than eyeballing. However, this is not what we observed in our present study: eyeballing resulted in MIB-1-LI read-outs that deviated much less from a central mean value than the counted data. Eyeballing is typically done at a smaller magnification than counting, making it easier to integrate slight locoregional variations and to arrive at more consistent average values. The smaller the field of interest, the higher the variability of computer-assisted MIB-1 LI values [26]. Also, counting may so occupy our brains that we may be less receptive for other important information. Those who were in the audience will remember the video clip shown at the 94^th^ annual meeting of the United States and Canadian Academy of Pathology in San Antonio, where we were all so busy counting how often a ball was bounced back and forth between the players of the black team that most of us failed to notice the huge black gorilla that slowly walked across the scene. Even though the fact that eyeballing yields more reliable MIB-1-LI results than counting may appear counter-intuitive at first sight, there exists no similar study in the literature that suggests that this should be different.

*… not improved by efforts to standardize what exactly are MIB-1 positive nuclei and where and how to count them.* After having failed to identify one or several confounding factors that could explain the high interobserver variability in MIB-1-LI results, we asked whether we had divergent notions of how a MIB-1-positive nucleus looks like. After all, there exists a spectrum of immunostaining intensities in MIB-1 immunohistochemistry, ranging from homogeneously dark-brown dots all the way down to lightly speckled nuclei. For this reason, we asked the participating pathologists to mark on an “intensity plate” to identify positive nuclei. No need to calculate percentages, just count. Some of us counted considerably fewer nuclei than others, obviously considering only the very dark nuclei as positive while the rest of us counted also the slightly stained nuclei as positive, as proposed by several authors [7], [17], [24]. We then reasoned that those colleagues who counted only the very dark nuclei should also be those who had under-estimated MIB-1-LI in our interobserver correlation analysis and vice versa. So, we plotted the number of positive nuclei counted on the “intensity plates” against the MIB-1-LI read-outs, but found no significant correlation suggesting that the interobserver variability could not be explained by different ideas of what counts as how a MIB-1 positive nucleus should look like.

In a similar study on paediatric sarcomas, Molenaar and co-workers arrived at the conclusion that a major part of the variability of MIB-1-LI remains unexplained, and suggested two ways to improve interobserver reliability: (i) systematic training (i.e. to standardize the tumor areas in which to look) and (ii) a mathematical correction for “personal bias” [27]. In the third step of our study we aimed to test these two avenues. We reasoned that through intensive intellectual confrontation with the subject matter (i.e. discussions of the results in the working group and at the annual conference of our society) and through improved standardization, we could obtain more consistent MIB-1-LI results. In analogy to the well-publicised DAKO plates depicting typical images of the three degrees of Her2/*neu* positivity (+, ++, +++), we distributed to the participating pathologists the six panel “intensity” plate on which we had circled all those nuclei that we had jointly defined as MIB-1 positive. Four months after the interobserver correlation study on the three moderately differentiated carcinomas (step 2), we sent the same three G2 carcinomas to the 15 pathologists again for MIB-1-LI reading. In addition, three new carcinomas (G1, G2, and G3) were sent included. As proposed by Molenaar and co-workers [27], we wanted to use the MIB-1-LI read-outs on these three new cases to calculate for each pathologist a “correction factor” (individual result/group mean) and test whether the interobserver variability of the three initial G2 carcinomas could be reduced by this simple mathematical manoever. However, the kappa values in this third round were even lower than in round two and we even obtained negative Kappa values, suggesting that our interobserver consistency was worse than throwing dice. Also, each individual pathologist's intra-observer consistency between MIB-1-LI results obtained initially and three months later on the same three tumors was scarcely better than throwing dice. Taken together, these findings do not support the proposition that interobserver variability can be reduced by prior efforts to obtain consensus regarding methods and appropriate interpretation of staining positivity [27], [28].

We hence have to assume that our capacity to reproducibly identify the percentage of MIB-1 positive tumor cells is likely governed by factors that reside in the largely undiscovered realm of cognitive psychology (affected by experience, expectation, bias, etc…) and cannot easily be improved by intellectual efforts to standardize the read-out technique [12], [29], [30]. For practical purposes, if a G2 carcinoma is read out by a particular pathologist as 24%, does that reliably mean that the patient will benefit from chemotherapy? Our study suggests that the same tumor might just as well have been signed out as 10% or 35% by another pathologist or by the same pathologist four months later. For the future, we might consider using computer-based image analysis, which has been found to yield more consistent MIB-1-LI results in GISTs [31], dysplasias in Barrett's esophagus [32], sarcomas [33], and breast carcinomas [23], [34]–[36]. However, beside variations during the digitization of slides, definition of positivity cut-offs and the reliable identification of “negative” cells (stroma vs. lymphocytes vs. tumor cells), computer-based image analysis harbors other distinct logistical problems (costs, time and manpower). This may eventually change with the availability of easily applicable open source techniques [37].

In conclusion, even though Kappa values have suggested that interobserver reliability of MIB-1-LI read-outs over a wide range of tumor differentiations is good, this promise appears not to hold true for those intermediately differentiated G2 carcinomas where oncologists would salute this marker as guidance for their treatment decisions in individual patients.
